# When Does Teacher Support Reduce Depression in Students? The Moderating Role of Students' Status as Left-Behind Children

**DOI:** 10.3389/fpsyg.2021.608359

**Published:** 2021-02-12

**Authors:** Wei Li, Wen yang Gao, Wei dong Fu

**Affiliations:** ^1^School of Education Science and Technology, Northwest Minzu University, Lanzhou, China; ^2^School of Management, University of Science and Technology of China, Hefei, China; ^3^Faculty of Artificial Intelligence in Education, Central China Normal University, Wuhan, China

**Keywords:** teacher support, depression, left-behind children, moderating role, elementary schools, middle/junior high schools

## Abstract

Teacher support (TS) makes students feel loved and cared for because they believe that their teachers will provide them with opportunities to make choices, support them in independent problem solving, and understand their inner feelings. High TS levels reduce depression and anxiety, thereby improving students' mental well-being. This cross-sectional study involved 3,573 students from 29 schools in 16 counties/cities of six provinces, namely, Guizhou, Hubei, Jiangxi, Shanxi, Sichuan, and Yunnan. The aim was to examine the impact of TS on students' level of depression. The results indicated that for children in elementary schools, their status as left-behind children (LBC) played a moderating role between TS and depression. The level of depression in non-LBC children decreased significantly with increases in TS, but the reduction for LBC children was not significant. For children in middle/junior high schools, their LBC status did not play a moderating role between TS and depression. TS was negatively correlated with the children's level of depression, but there was a significant positive relationship between their LBC status and depression. The theoretical and practical significance of the research findings were further discussed.

## Introduction

Left-behind children (LBC) refer to minors under 18 years of age (Luo et al., 2009; Su et al., 2013) who stay back in the countryside for 3 months or more when one parent (or both) is a migrant worker in the city (Fan et al., 2012). With the rapid development of China's economy, the surplus labor force in rural areas has migrated to the cities in search of employment. Due to the dual system with a well-defined urban–rural structure and the corresponding limitations in education, medical care, and other social resources, the vast majority of migrant workers leave their child(ren) in their hometowns and entrust them to the care of relatives or friends. This has led to the phenomenon of left-behind children (LBC), which is unique to China. Recent surveys reveal that there are nearly 61 million LBC in rural China, accounting for 58.1 and 21.9% of the total number of children in rural areas and the country as a whole, respectively (Wang and Liu, 2020). LBC face numerous psychological and behavioral issues because of long-term separation from their parents and the lack of effective supervision and care. Research on LBC, especially their mental health, is also increasing.

Compared to non-LBC (NLBC), LBC report higher risks of depression (He et al., 2012; Cheng and Sun, 2015) and anxiety (Zhao et al., 2014; Cheng and Sun, 2015), lower levels of hope and pro-social behaviors (Yang et al., 2013, 2014; Wang et al., 2017), poorer self-esteem (Luo et al., 2009; Tomsa and Jenaro, 2015; Xing et al., 2017), and other psychological problems (Ye et al., 2017). LBC have usually been found to have a deteriorated status of depression compared with NLBC (He et al., 2012; Xiao et al., 2019). A meta-analysis has shown that the detection rate of symptoms of depression among LBC was 28.9% (Xu et al., 2016).

### Teacher Support and Depression

The social support theory proposes that social support has both buffering effects and main effects (Cohen and Wills, 1985) on mental health, as social support is an important resource for individuals to obtain information, advice, help, and support. When individuals face mental stress, both social relationships and support enable them to better respond and deal with the situation psychologically and behaviorally. At the same time, their basic needs for relationships are met (House et al., 1988). Social support refers to individuals feeling loved and cared for, with the main sources being one's family, peers, and teachers (Camara et al., 2017).

Teacher support (TS) allows students to feel loved and cared for because they believe that their teachers will give them with opportunities to make choices, support them in independent problem solving, and understand their inner feelings (Cohen and Wills, 1985; Mageau and Vallerand, 2003). As a potential provider of assistance, teachers not only help students solve their psychological problems but also promote their mental well-being (Kim and Kim, 2013; Sisask et al., 2014). In fact, TS, rather than support received from family and friends, directly correlates with students' mental health (Cattley, 2004). The support that teachers provide to their students and the relationships among children affect many aspects of their growth, such as social adaptation (Shokeen, 2017), mental health (Shokeen, 2017), learning engagement (Roorda and Oort, 2011), and adaptation to school (Sun and Qiu, 2010; Longobardi et al., 2016; Koca, 2017). Based on the foregoing, the following research hypothesis was proposed: **H1**: There is a significant negative correlation between TS and depression.

### Moderating Role of Left-Behind Children Status Between Teacher Support and Depression

Many studies have confirmed the positive impact of TS on students' mental well-being. A study by Yu et al. (2016) found a significant negative correlation between students perceiving that their teachers provide autonomy support and their levels of anxiety and depression after 1 year. Such research conclusions further verify the main effect model of the social support theory, that is, social support plays a positive role regardless of the status of the external environment. An individual's developmental process is shaped by the combined effects of the external environment and their inherent characteristics. As such, it is necessary to consider the important factor of individuals' characteristics when examining the positive role of social support.

The absence of parents playing a long-term role in their lives causes LBC to develop distinct psychological characteristics, such as depression, anxiety, and poorer self-esteem. The main reason for such negative psychological characteristics may be LBC's failure to form good relationships with, and an attachment to, their parents. According to the internal working model of attachment theory, the pattern of interactions that characterize an individual's early attachment to their parents affects their view of themselves and others. This in turn affects how they interact with others (Bretherton, 1992). This theory focuses on the impact of attachment on individuals' patterns of cognitive and emotional responses. The expectations of self and others thus formed then affect their future patterns of interpersonal responses (Tao, 2011).

LBC are more likely to form negative expectations of others and have difficulties feeling social support from other people compared with NLBC. Therefore, no matter how high the level of social support given by their teachers, the inhibitory effect of that support on the level of depression may not be obvious. NLBC are nurtured by their good relationship with, and attachment to, their parents, making them more likely to form positive expectations of themselves and others. Correspondingly, they can easily feel the support from their teachers, meaning that TS has a more obvious effect in inhibiting their depression. On this basis, the following research hypothesis was proposed: H2: The LBC status of students may play a moderating role between TS and depression. For LBC, TS has no significant relationship with students' depression; for NLBC, TS has a significant negative relationship with students' depression.

## Research Method

### Participants

All the research processes were reviewed and approved by the Survey and Behavioral Research Ethics Committee in the author's university. A convenience sampling method was used to select 29 schools in 16 counties/cities of six provinces—Guizhou, Hubei, Jiangxi, Shanxi, Sichuan, and Yunnan—to conduct a cross-sectional study. It involved a total of 3,573 students in elementary and middle/junior high schools. Written informed consent was obtained from the schools, as well as the parents or guardians of students who voluntarily participated in the survey. The criteria for the selection of participants are as follows: Students from the 4th−9th grades who were willing to participate and had normal Chinese reading and comprehension abilities. The criteria for exclusion are the following: students with a congenital intellectual disability, brain injury (trauma or severe organic changes), or previous history of mental illness. During the survey, the researcher first explained the purpose, significance, and voluntary and confidential principles of the survey to the participants. Next, the investigators explained the contents of the survey questions individually. The survey questionnaires were distributed and retrieved on the spot.

Participants who were elementary school students were 1,241 comprising 585 boys (47.1%), 654 girls (52.7%), and two (0.2%) who did not state their gender. Participants who were middle/junior high school students were 2,332 comprising 1,035 boys (44.4%), 1,292 girls (55.4%), and five (0.2%) who did not state their gender. In terms of the spatial distribution of schools involved, 159 (12.8%), 596 (48%), and 486 (39.2%) participants in elementary schools were from counties/cities, townships, and villages, respectively. The spatial distribution for participants in middle/junior high schools was 622 (26.7%), 1,514 (64.9%), and 196 (8.4%), respectively. For participants in elementary schools, 28 (2.3%), 506 (40.7%), and 706 (56.9%) were in the 4th, 5th, and 6th grades, respectively. For participants in middle/junior high schools, 530 (22.7%), 1,263 (54.1%), and 540 (23.2%) were in the 7th, 8th, and 9th grades, respectively.

For the family structure of participants in elementary schools, 1,066 (85.9%) and 158 (12.7%) were from nuclear and single-parent families, respectively; 12 (1.0%) were orphans, and five (0.4%) did not state the related information. For that of participants in middle/junior high schools, the breakdown was 2,096 (89.9%), 220 (9.4%), 12 (0.5%), and 4 (0.2%), respectively. For participants in elementary schools, their parents' educational qualifications were as follows: 35 (2.8%), 42 (3.4%), 218 (17.6%), 556 (44.8%), 338 (27.2%), 32 (2.6%), 20 (1.6%) of the fathers completed college and above, vocational college, senior high school, middle/junior high school, elementary school, had no formal education, and did not state the related information, respectively. The breakdown for the mothers was 30 (2.4%), 37 (3.0%), 230 (18.5%), 463 (37.3%), 350 (29.6%), 72 (5.8%), and 59 (4.8%), respectively. For participants in middle/junior high schools, 80 (3.4%), 71 (3.0%), 244 (10.5%), 1,083 (46.4%), 728 (31.2%), 88 (3.8%), and 38 (1.6%) of the fathers completed college and above, vocational college, senior high school, middle/junior high school, elementary school, had no formal education, and did not state the related information, respectively. The breakdown for the mothers was 52 (2.2%), 60 (2.6%), 246 (10.5%), 956 (41%), 691 (29.6%), 252 (10.8%), and 75 (3.2%), respectively.

### Research Tools

#### Children's Depression Inventory

This study used the children's depression scale developed by Ren and Tang (2014) to measure the children's' level of depression. This scale was adapted from the depression scale compiled by Radloff (1991) and is suitable for measuring the general depressive emotional state of adolescents. The scale included six questions, for which the children had to indicate how often they experienced each symptom in the past month. The options 0, 1, 2, 3, and 4 represented never, once a month, two to three times a month, two to three times a week, and almost every day, respectively. The α coefficient of the scale used was 0.72.

#### Teacher Support

A TS scale with three questions was constructed. The children had to answer each question based on actual conditions in the recent semester. The specific questions were: “The teacher provides you with the opportunity to raise queries and answer questions in class,” “When you do not understand something during your studies, you will get guidance or help from the teacher,” and “Do you feel that your teacher cares about you?” The options 0, 1, 2, and 3 represented never, occasionally, often, and always, respectively. The α coefficient of the scale used was 0.59. The maximal reliability was 0.62 (χ^2^ = 0, *df* = 0, CFI = 1.0, TLI = 1.0, RMSEA = 0, SRMR = 0).

#### Left-Behind Children

The term LBC has become very well-known in China, rather than being a strictly academic concept. Hence, the children were directly asked, “Are you an LBC?” to obtain the relevant statistics. The variable “whether left-behind children” was transformed into dummy variables. The children indicated 0 or 1, which represented NLBC and LBC, respectively. For children in middle/junior high schools, 540 (23.2%) and 1,791 (76.8%) were LBC and NLBC, respectively; one did not state the relevant information. Among those in elementary schools, 422 (34%) and 816 (65.8%) were LBC and NLBC, respectively; three (0.2%) did not state the relevant information.

### Data Analysis

First, the expectation-maximization (EM) algorithm was used to fill in the missing values. The SPSS 25.0 software was then used for calculating descriptive statistics of the data, independent-sample *t*-tests, Pearson product-moment correlation analysis, and multiple linear regression analysis. Additionally, the same software was used to perform the Harman's single-factor method to assess the severity of common method variation (CMV).

## Results

### Test for Common Method Variation

The self-reporting method used in this study to collect data from the participants might result in CMV. Thus, the testing process had to be strictly controlled: (i) It was emphasized that the questionnaire results would only be used for academic research, and all information provided was kept strictly confidential. (ii) The questionnaire was filled in by the participants anonymously. The Harman's single-factor test was used to test the data for CMV (Podsakoff et al., 2003), while unrotated exploratory factor analysis was performed on the questions in the two scales for depression and TS. The results showed that there were two factors with an initial eigenvalue > 1. The variance explained by the first factor was 34.219%. This was less than the critical value of 40%, which indicated that CMV in this study was not significant (Xiong et al., 2012).

### Correlation Analysis Between Depression, Teacher Support, and Left-Behind Children Status

The average scores of NLBC middle school students for teacher support and depression were 5.94 (SD = 1.63) and 9.85 (SD = 4.9), LBC students were 6.0 (SD = 1.63) and 10.40 (SD = 5.14). The average scores of NLBC primary school students for teacher support and depression were 6.52 (SD = 1.58) and 8.15 (SD = 4.78), LBC students were 6.50 (SD = 1.59) and 8.49 (SD = 4.73). Values of means, standard deviations, and correlation coefficients are presented in Table 1. There was a significant negative correlation between TS and depression for students in all grades. However, the correlation of LBC status with depression and TS was low (*r* value ranged from −0.008 to 0.046), and included both positive and negative correlations.

**Table 1:** Descriptive statistics between the study variables and related correlation matrices.

**Variable**	**Middle/junior high schools**		**Elementary schools**	
	**Depression**	**TS level**	**Depression**	**TS level**
Depression	1		1	
TS level	−0.191^**^	1	−0.189^**^	1
LBC status	0.046^*^	0.016	0.034	−0.008
Mean	9.977	5.954	8.264	6.512
Standard deviation	4.963	1.634	4.767	1.585

* p < 0.05,

** *p < 0.01. TS, teacher support; LBC, left-behind children*.

### Analysis on Whether Left-Behind Children Status Played a Moderating Role Between Teacher Support and Depression

We adopted the test procedure proposed by Muller et al. (2005) to analyze the moderating role of LBC status between TS and depression. After controlling variables such as the students' gender, education qualification of their parents, and location of their schools (counties/cities, townships, or villages), it was found that the interaction term between TS and LBC status had a significant effect on depression for the group of students in elementary schools (Tables 2, 3). However, the effect from the interaction term to depression for students in middle/junior high schools was not significant. In other words, LBC status played a moderating role between TS and depression for students in elementary schools, but did not play such a role for students in middle/junior high schools.

**Table 2:** Analysis on whether LBC status had a moderating role between perceived TS and depression for students in elementary schools.

**Variable (elementary schools)**	**Step 1**		**Step 2**		**Step 3**	
	**(Dependent variable for Steps 1-3: depression)**					
	**B (SEB)**	***t***	**B (SEB)**	***t***	**B (SEB)**	***t***
**Control variables**						
(Constant)	8.171 (0.944)	8.654^***^	7.671 (0.938)	8.179^***^	7.749 (0.931)	8.322^***^
Gender	−0.09 (0.279)	−0.323	0.065 (0.275)	0.236	0.005 (0.274)	0.017
Father's educational qualification	−0.19 (0.16)	−1.186	−0.137 (0.158)	−0.867	−0.146 (0.157)	−0.931
Mother's educational qualification	−0.003 (0.151)	−0.023	0.021 (0.149)	0.141	0.03 (0.147)	0.205
Location of school	0.316 (0.221)	1.43	0.33 (0.221)	1.498	0.335 (0.219)	1.532
**Independent variables**						
TS			−0.903 (0.137)	−6.598^***^	−0.895 (0.136)	−6.586^***^
LBC status			0.091 (0.139)	0.654	0.085 (0.138)	0.614
LBC status × TS					0.584 (0.136)	4.301^***^
*R*^2^	0.004		0.04		0.056	
*Δ R*^2^	0.004		0.036^***^		0.015^***^	
*F*	1.183		8.144^***^		9.729^***^	

*** *p < 0.001. TS, teacher support; LBC, left-behind children. Data for TS and LBC status were subjected to centralized treatment before being entered into the equation, while their interaction term was the product of the centralized variables. Therefore, only results that were non-standardized were reported (Fang et al., 2015). The same procedure was applied to obtain the results stated in the next table*.

**Table 3:** Analysis on whether LBC status had a moderating role between perceived TS and depression for students in middle/junior high schools.

**Variable (middle/junior high schools)**	**Step 1**		**Step 2**		**Step 3**	
	**(Dependent variable for Steps 1–3: depression)**					
	**B (SEB)**	***t***	**B (SEB)**	***t***	**B (SEB)**	***t***
**Control variables**						
(Constant)	10.706 (0.683)	15.685^***^	10.544 (0.675)	15.62^***^	10.544 (0.675)	15.617^***^
Gender	−0.034 (0.212)	−0.16	−0.031 (0.208)	−0.149	−0.032 (0.208)	−0.153
Father's educational qualification	−0.129 (0.128)	−1.011	−0.091 (0.126)	−0.722	−0.091 (0.126)	−0.721
Mother's educational qualification	−0.154 (0.123)	−1.254	−0.125 (0.12)	−1.039	−0.126 (0.121)	−1.047
Location of school	−0.053 (0.157)	−0.388	−0.041 (0.156)	0.261	−0.043 (0.156)	0.274
**Independent variables**						
TS			−0.895 (0.103)	−8.654^***^	−0.895 (0.103)	−8.654^***^
LBC status			0.207 (0.105)	1.978^*^	0.059 (0.104)	1.983^*^
LBC status × TS					−0.045 (0.103)	−0.567
*R*^2^	0.003		0.037		0.037	
Δ*R*^2^	0.003		0.034^***^		0	
*F*	1.652		14.217^***^		12.228^***^	

* p < 0.05,

*** *p < 0.001. TS, teacher support; LBC, left-behind children*.

**Figure 1:**
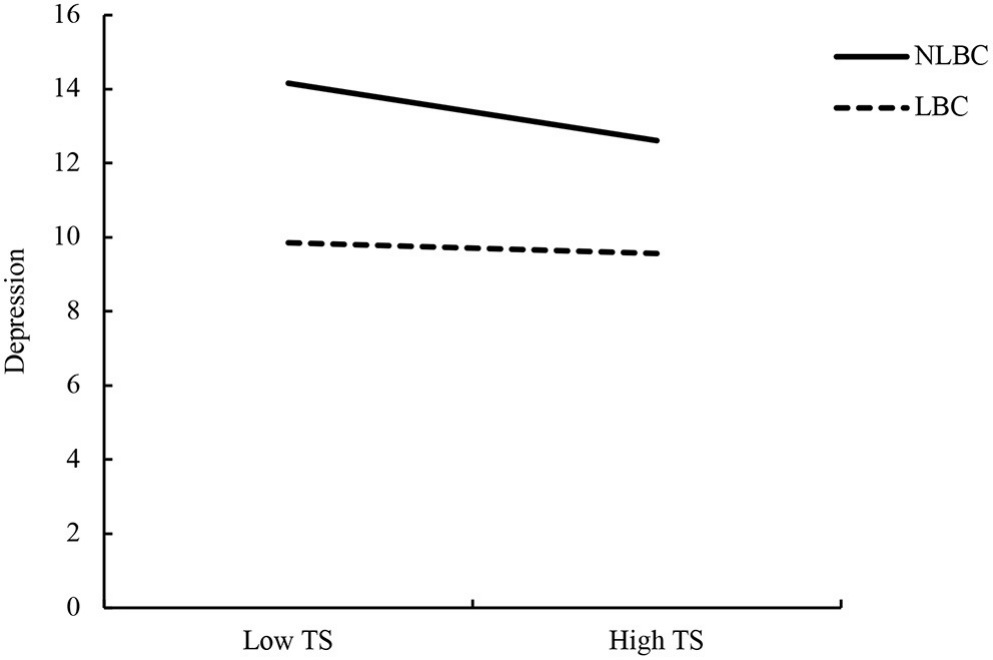
Moderating role of left-behind children (LBC) status on the relationship between teacher (TS) and depression of students in elementary schools.

A simple slope test was performed to further explore the moderating role that LBC status had between TS and depression of students in elementary schools. The results indicated that TS had no significant effect on the depression of LBC students (β = −0.036, *t* = −0.73, *p* = 0.466). However, the effect of TS was significant for NLBC students (β = −0.264, *t* = −7.802, *p* < 0.001). The interaction diagram (Figure 1) intuitively reflects the moderating role that LBC status had on the relationship between TS and depression of students in elementary schools.

LBC status did not play a moderating role between TS and depression of students in middle/junior high schools. Further analysis on the effect of TS on depression showed that the correlation between these two variables was significantly negative for both LBC and NLBC. Specifically, the effect of TS on depression was significant for LBC students (β = −0.219, *t* = −5.2, *p* < 0.001) and NLBC students (β = −0.183, *t* = −7.88, *p* < 0.001).

## Discussion

### Moderating Role of Left-Behind Children Status Between Teacher Support and Depression of Students in Elementary Schools

Analysis of students in elementary schools revealed that LBC status played a moderating role between TS and depression. For elementary school students, hypotheses H1 and H2 were supported. Although the TS level had no significant impact on LBC students, the correlation between the levels of TS and depression was significantly negative for NLBC students. These results were in line with the ecosystem theory. The family is the most important micro-level system during an individual's growth process (Bronfenbrenner, 2005), and family support is the most important support resource for children. Compared with NLBC, LBC perceived a lower level of parental care. The latter's needs in the family domain are, to a greater extent, not being met, and their desire for parental love is stronger (Chang and Xia, 2008; Fan et al., 2012).

Although receiving and making use of social support are directly related to lower levels of depression (Camara et al., 2017), the moderating role of TS in alleviating depression in LBC has limited effectiveness because their parents, an important factor, are missing. The lack of parental support had a significant impact on predicting depressive symptoms (Stice et al., 2004). In other words, TS is one aspect with regard to the mental health development of LBC, but more important is establishing good parent–child communication and interaction. For example, after Fu et al. (2016) established a dozen experimental sites in Xianning City of Hubei Province and the Liangshan Yi Autonomous Prefecture in Sichuan Province, they harnessed information technology for video calls between children and their parents who are migrant workers. The calls were made two to three times a week at fixed times. Parent–child contacts through this televisual medium effectively eliminated the sense of distance and produced excellent results.

### Left-Behind Children Status Played No Moderating Role Between TS and Depression of Students in Middle/Junior High Schools

Through analysis of the group of students in middle/junior high schools, it was found that their LBC status did not play a moderating role between TS and depression. However, there was a significant correlation between TS and LBC status with depression. For middle school students, hypotheses H1 was supported, and H2 was rejected. Previous research found that LBC experience far more negative psychological emotions such as depression and anxiety than NLBC (He et al., 2012; Xu et al., 2016), whereas positive teacher–student relationships and TS reduce symptoms of anxiety and depression in adolescents (Cao, 2006; Krane et al., 2017; Shokeen, 2017). TS for middle/junior high school students had a significant impact on reducing their depression because they might be able to make more objective evaluations of their teachers at this stage.

Cohen and Wills (1985) pointed out that different needs have to be met by dissimilar types of support. Teachers are often able to provide students with the necessary support information-wise and some problem-solving strategies or suggestions. They also play the role of mentors in schools, which makes students more likely to accept the support that they provide. Therefore, TS significantly reduced depression in students who were students in middle/junior high school. Depression levels in LBC might have been significantly higher than in NLBC because of the rebellious psychological characteristics of students at that age. They are detached in their relationships with their parents in terms of emotions, behaviors, and perspectives, but their way of thinking, self-awareness, and other psychological aspects have not really reached the mature level of adulthood, meaning that they still need the support and assistance of their parents. Unfortunately, parent–child emotional exchanges are limited because their parents are working elsewhere, such that students are more likely to become depressed when they feel troubled (Camara et al., 2017). Some studies have found that TS is more likely to be in the form of providing students with advice and related skills (Kim and Kim, 2013; Sisask et al., 2014; Fan and Lu, 2020). In contrast, emotional support from one's family, especially the mother, reduces stress substantially (Camara et al., 2017). When facing stressful incidents, LBC are more likely to experience emotional problems because of their lack of social support resources.

Unlike in students from elementary schools, TS had a significant effect on reducing depression in students in middle/junior high schools. However, this effect was not affected by the moderating role of LBC status. This could be related to the characteristics of the stage of development in an individual's self-consciousness. Self-consciousness is better developed in students at that age, and they are more independent and demand the right to enjoy their independence. They consider the care given by their parents regarding their lives and emotional aspects to be obstacles to their independence (Lin, 1995). Elementary school students are different in that they have lower levels of self-consciousness. Self-evaluation is primarily obtained from social support, in which their parents play the most important role, rather than their friends and teachers (Lin, 1995).

This situation might also be related to the beneficial interpretations, that is, the extent to which individuals approved the behaviors of their parents and believed that the latter are acting for the individuals' good (Camras et al., 2012). With increasing age, children's cognitive evaluation of their parents being migrant workers may change. The beneficial interpretations given by the parents of LBC can facilitate the latter's social adaptation, thereby reducing the occurrence of depression and problematic behaviors in them (Tavassolie et al., 2016; Yang et al., 2016). This better explains the difference in the moderating role of LBC between students in elementary vs. middle/junior high schools when it came to TS and depression.

## Research Implications and Limitations

The implications of this research lies in the following: (1) Through the test of the effect from teacher support to students' depression emotion, the study further confirms the universal existence of buffering effects and main effects of social support theory, which will help us to more objectively and comprehensively evaluate the effect of social support on mental health. (2) The study explains the boundary conditions of the positive role of teacher support and provides enlightenment for educators to effectively apply the positive role of teacher support in their work. Even while emphasizing the impact of TS on students' mental well-being, attention must also be paid to the characteristics of different groups at various ages. For example, from the perspective of a beneficiary explanation, it is critical to note the children's cognitive evaluation of the fact that their parents are migrant workers. If they think that their parents' actions are for the purpose of giving them a better life, their sense of abandonment and other negative emotions generated by their parents' departure will be ameliorated. Facing NLBC of elementary schools, we should play the role of teacher support to promote students' mental health. However, facing LBC of elementary schools, we must not only give play to teacher support but also emphasize the participation of family support.

This study had some limitations. First, the cross-sectional survey method data limits us to infer causality. In the future, longitudinal designs may enhance our understanding about the causal relationships between TS and depression of students. Second, we focus on the relationships between TS and depression of students; however, there could be other confounding variables in this model, such as some characteristics of the child (such as the sense of self-efficacy) or school performance (success vs. failure), both parents or one parent went out to work. We know that these aspects may have aspects on the school well-being of the child. It would be useful for future work to examine these intervening variables. Last, the study only focused on the role of TS for elementary and middle/junior high school students, but did not take into account other supporting factors under social support, such as peer support and family support. For further verification, the scope of investigation should be expanded in future research.

## Data Availability Statement

The original contributions presented in the study are included in the article/Supplementary Material, further inquiries can be directed to the corresponding author/s.

## Ethics Statement

The studies involving human participants were reviewed and approved by Research Ethics Committee, School of Education Science and Technology, Northwest Minzu University. Written informed consent to participate in this study was provided by the participants' legal guardian/next of kin.

## Author Contributions

WL: writing - original draft and formal analysis. WG: data curation, writing-review, and editing. WF: conceptualization, methodology, and investigation. All authors contributed to the article and approved the submitted version.

## Conflict of Interest

The authors declare that the research was conducted in the absence of any commercial or financial relationships that could be construed as a potential conflict of interest.
